# Crosslinking-Dependent Drug Kinetics in Hydrogels for Ophthalmic Delivery

**DOI:** 10.3390/polym14020248

**Published:** 2022-01-08

**Authors:** Nicole Mortensen, Parker Toews, Jeffrey Bates

**Affiliations:** Department of Materials Science and Engineering, University of Utah, 122 Central Campus Drive, Room 304, Salt Lake City, UT 84112, USA; nmortensen01@gmail.com (N.M.); parker.m.toews@utah.edu (P.T.)

**Keywords:** hydrogels, crosslinking, ophthalmic drug delivery, optimization study

## Abstract

Drug-diffusion kinetics in 2-hydroxyethyl methacrylate hydrogels were studied as a function of the crosslinking density and porosity. By varying the concentration of the crosslinker, tetraethylene glycol dimethacrylate, we demonstrated how the release of Timolol maleate could be optimized to allow for efficient drug delivery. FTIR and spectrophotometry supplied optical inferences into the functional groups present. By studying the swelling and degradation of hydrogels, supplemented with drug-release kinetics studies, the relationship between these two tenets could be formulated.

## 1. Introduction

The human eye is debilitated by various diseases, including glaucoma. In 2010, it was suggested that 60.5 million people worldwide would suffer from open-angle glaucoma (OAG) or angular-closure glaucoma (ACG). Modeling predicted that there would be 79.6 million people worldwide with some form of glaucoma. Furthermore, there exists distinct disproportionality of glaucoma affecting women and Asians [1]. A distinction present in glaucoma is that 50% of those with the disease are not aware of having it. This is due to the inherent lack in the presentation of symptoms of the disease [2]. Glaucoma is an eye disease which creates an increase in interocular pressure (IOP), which can lead to vision loss or, in extreme cases, blindness [3]. A medication used to treat glaucoma is Timolol maleate, or Timolol. It falls under the categorization of a beta-blocker and treats glaucoma through reducing the IOP by reducing the aqueous humor secretion in the body [4].

Hydrogels are identified scientifically as a hydrophilic polymer that is biocompatible and have a determined stimuli-response [5,6,7,8,9,10,11]. Their mechanical and chemical properties have made them available to a variety of biomedical applications; not limited to tissue engineering, actuators, sensors, and drug-delivery devices [5,6,12,13,14,15,16]. Hydrophilic functional groups attached to the backbone give the ability for hydrogels to absorb water [17] (pp. 18–23). Furthermore, the presence of physical or chemical crosslinking, hydrogen, and covalent bonds allows for the insolubility of these materials [18] (pp. 57–64). Modern contact lenses are classified as a hydrogel, allowing a level of permeability of oxygen to the eye, which is dictated by the water content of the lens [19] (pp. 24–43).

**Figure 1:**
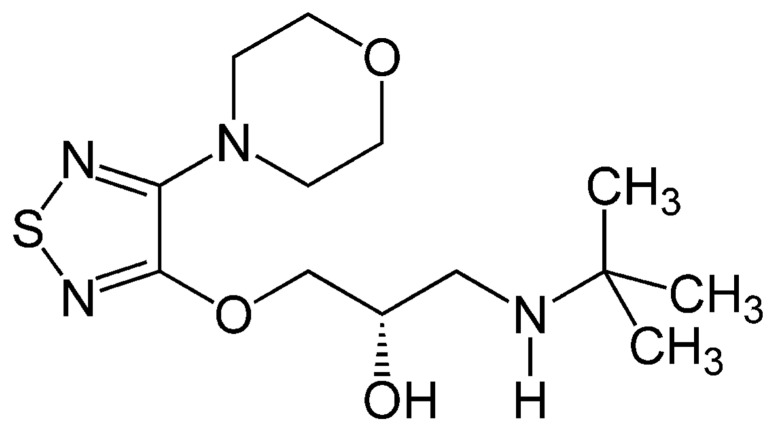
Demonstrates the monomer structure of the Timolol medication. Sites available allow for increased drug-release efficiency, due to the hydrogen-bonding sites present [36].

Molecular imprinted (MIP) hydrogels were studied for their swelling properties [20,21]. Their swelling allows for the controlled release of ocular medication from the polymer matrix to the ocular tissue. MIP samples were compared with their non-imprinted (NMIP) counterpart to determine drug-release differences between the two composition techniques. This is preferred to current drug delivery methods which utilize biodegradation of the hydrogel to control the release of medication. It is identified, however, that toxic substances can be inadvertently released to the treatment site, due to the contained functional groups present [22,23]. Challenges related to the degradation responses of hydrogels can be remediated by leveraging chain entanglement over covalency in crosslinking, thus improving the tunability [24] (pp. 1149–1161). Molecular imprinting is accomplished by mixing the target molecule into pre-polymerized solution. As polymerization occurs, the target molecule—Timolol, in this case—is encapsulated into the final polymer matrix [25]. The target molecule can exhibit covalent or non-covalent bonding behavior to the rest of the polymer. Drugs such as Timolol exhibit non-covalent bonding with the polymer matrix due to the weak chemical interactions found in covalent bonding [26,27]. Timolol, specifically, makes for a suitable template for MIP, due to the multiple hydrogen-bonding sites made available [28]. These sites allow for the recognition of Timolol within the polymer structure, such that further additions of Timolol will bind to the pre-existing sites in the structure [29,30,31]. During the imprinting reaction, the polymeric chains will organize around the imprinted molecule, allowing for this recognition [25,30,32,33]. Timolol’s structure (shown in Figure 1, below) noticeably contains hydrogen bonds. These hydrogen bonds serve as the best bonding platform, as they pre-exist in NMIP polymer construction [34,35].

Furthermore, having this response in the hydrogel makes for a reversible system [37]. This reversibility allows for the release and uptake of medication from the hydrogel system to, in this case, the ocular tissue.

This paper seeks to combine the knowledge of ophthalmology with materials science and engineering to create an efficient and dependable drug-delivery vehicle for glaucoma patients. Specifically, this means having a system which can promote the hydrogen bonding of Timolol into the polymer matrix, a high transparency, and low swelling. The current use of contact lenses worldwide, being near 41 million in the United States alone, seeks to promote the currently low patient adherence to glaucoma treatment [38] (pp. 865–870). Through NMIP and MIP hydrogel testing, it is shown how the drug delivery of HEMA-backbone hydrogels depend on the crosslinking density of the material. 

## 2. Materials and Methods

Ammonium persulfate (APS), 2-(dimethylamino) ethyl methacrylate (DMAEMA), ethylene glycol (EG), 2-hydroxyethyl methacrylate (HEMA), tetraethylene glycol (TEGDMA), *N*,*N*,*N*′,*N*′-tetramethylethylenediamine (TEMED), and Timolol were purchased from Sigma-Aldrich (St. Louis, MO, USA) and used as received for synthesis. Off-shelf contact lenses (JJ1) were obtained from Johnson and Johnson (New Brunswick, NJ, USA) and used as received for testing.

Hydrogels were synthesized by thermally initiated free-radical polymerization. Two compounds were combined, namely A and B. Compound A consisted of an initiator (APS) and a solvent (EG). This was mixed individually for 10 min to prevent the development of clumps or cloudiness in the final product. Compound B consisted of the backbone (HEMA), a pH-sensitive monomer (DMAEMA), a crosslinker (TEGDMA), and a catalyst (TEMED). For non-imprinted (NMIP) samples, Compounds B and A were combined for 15 s and left to polymerize at 5.0 °C for 24 h. For imprinted (MIP) samples, Compound B was imprinted with 25 μL of Timolol, which was left to imprint at 5.0 °C for 24 h. Once imprinting was completed, Compound B was mixed into Compound A and left to polymerize at 5.0 °C for 24 h. This temperature and time were selected to slow the reaction down, allowing for both uniformity in the final product and improved optical properties.

For this experiment, a series of seven formulations were derived with varying crosslinker concentrations. Amounts of initiator, solvent, backbone, pH-sensitive monomer, and catalyst remained the same: 1.3 mg (0.08%), 273.9 μL (26.2%), 218.7 μL (68.4%), 36.45 μL (3.59%), and 2.3 μL (0.31%) respectively. The amount of crosslinker varied from 20 to 50 μL, as demonstrated for samples in Table 1.

Whilst maintaining the number of other components existing in the final volume, and varying the TEGDMA volume, the Timolol volume was maintained at 1.5 μL, or a mass fraction percentage of 0.05%. 

Timolol solutions were constructed in the medically prescribed concentrations of 0.025% and 0.05% [11]. As Timolol originates in powder form, 0.75 and 1.5 mg, respectively, were measured out and combined with 30 mL of deionized (DI) water. The solution was then mixed and kept covered at 5.0 °C for future use.

A Varian 3100 (Walnut Creek, CA, USA) Excalibur Fourier-transform infrared spectroscopy (FTIR) was utilized in this study to identify the chemical structure of the synthesized samples, as well as the JJ1 samples. Absorbance measurements were conducted by utilizing attenuated total reflection (ATR) with a KBR crystal. This ATR setup produced peaks between 1900 and 2400 cm^−1^ and at 3400 cm^−1^ that are not attributed to the polymers.

Using a PerkinElmer (Waltham, MA, USA) LAMBDA 950 UV–Vis spectrophotometer, we determined the transmittance of samples to determine the optical properties of the materials. In this manuscript, this is classified as being the transmittance of the system, on top of the absorbance for determining the drug kinetics. 

Absorbance data from spectrophotometry were utilized to determine the concentration of Timolol through the Beer–Lambert Law. Herein, A is the absorbance, ε is the molar absorption coefficient, l is the path length, and c is the solution concentration. This law explicitly states that the absorbance of the solution is directly proportional to the concentration when the molar-absorption coefficient and path length remain constant. The Beer–Lambert Law, in formula form, is stated in Equation (1).

A = εlc(1)

Swelling ratios were determined by measuring the initial mass of the samples, which were kept consistent at 0.06 g for all samples, and the sequential swelled mass for three days. From there, by noting these two weights, it was plausible to determine the swelling ratio through Equation (2), as shown below, where S is the swelling ratio, m_s_ is the mass of the swelled hydrogel, and m_d_ is the dry mass of the hydrogel.

S = m_s_ − m_d_/m_d_ (2)

## 3. Results

### 3.1. Timolol Standards

By varying the concentrations of Timolol in spectrophotometry, it was plausible to model a calibration curve. Concentrations of 0.005, 0.01, 0.025, and 0.05 mM were tested at the lowest spectrophotometer setting, 325 nm. This revealed a linear behavior, following Equation (3).

y(x) = 7.9878x + 0.4068(3)

This linear equation follows similarly to the Beer–Lambert law, making it a statistically confident model for determining the concentration of Timolol in samples presented. The equation follows such that y exists as the term expresses the absorbance collected from the spectrophotometer and x represents the concentration of Timolol, in mM.

### 3.2. Optical and Physical Properties

**Figure 2:**
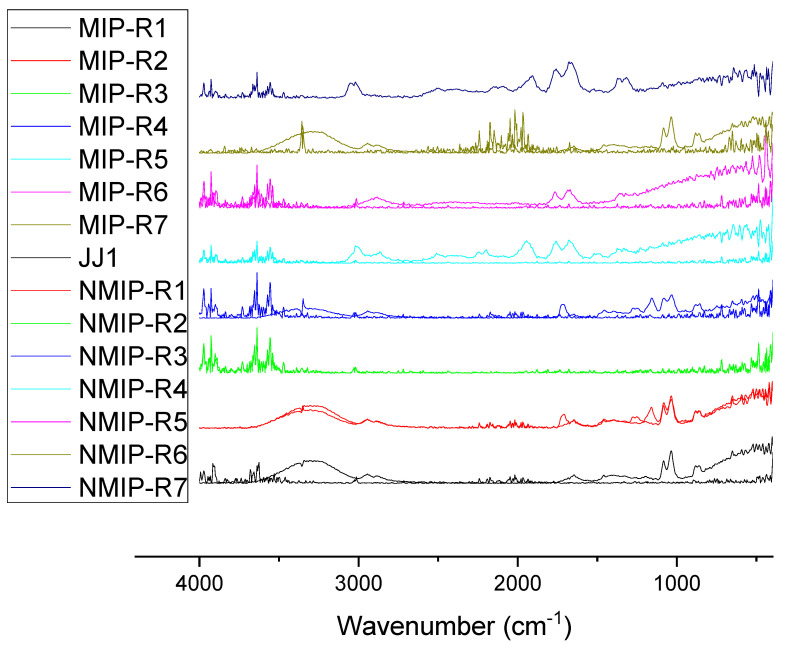
Demonstrating the FTIR spectrum for various samples compared to JJ1. *X*-axis here represents the wavenumber in inverse cm. The *y*-axis in this figure is not demonstrated, due to the samples being tiled above one another for easier appreciation of the dataset.

Figure 2, below, shows the FTIR–ATR results for NMIP and MIP samples.

When utilizing this data, it is valuable to identify the significant functional groups present in the spectra. Herein, one can note the following peaks in the spectra: alkene (C=C), amine (C–N), ether (C–O–C), and alkane (C–H). These correspond to wavenumbers of ~1505, 1317, ~1250, and ~659 cm^−1^, respectively, with the alkene group being the most prevalent in the majority of samples. 

**Figure 3:**
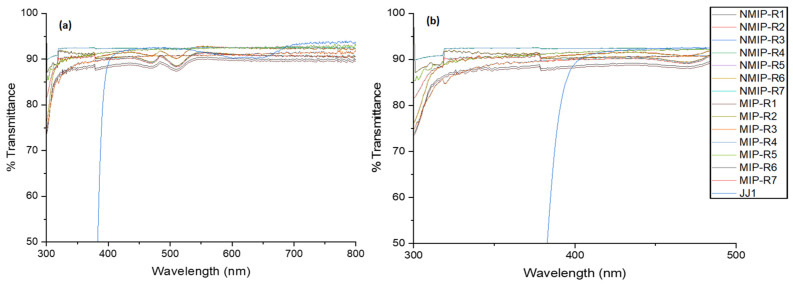
(**a**) Demonstrating the % transmittance for all samples utilized in this study, noting that % transmittance is presented in the *y*-axis, with wavelength presented on the *x*-axis. (**b**) Demonstrating the performance of the hydrogels in the slight UV spectrum, compared with on-market contact lenses.

Looking into the optical properties further, we found that it was plausible to investigate the overall transmittance of all samples, both NMIP and MIP, considered in this study. These results are used to corroborate the previously discussed FTIR measurements, but they also motivate the performance of the hydrogels in later sections of this paper. This is demonstrated below by Figure 3.

Figure 3 shows the transmission of all hydrogel samples constructed and shows interesting performance disparities, which are further discussed in Section 4. From noting the optical properties of the system, it is plausible to transition into the swelling and drug kinetics of the material.

### 3.3. Swelling Kinetics

**Figure 4:**
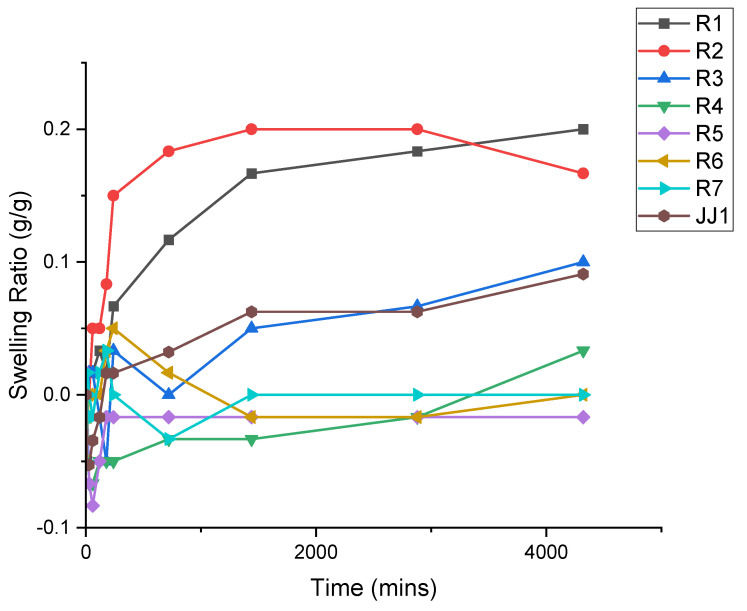
Demonstrating the swelling ratios (g/g) for NMIP samples over a period of three days. The *y*-axis demonstrates the swelling ratio, which is compared to the *x*-axis in units of minutes.

Through exploring the swelling kinetics in the hydrogel system, it was plausible to determine the ability of the hydrogel to remain consistent volumetrically when utilized as a prescription contact lens. For the purposes of this section of the study, only NMIP compositions were studied and are designated as simply “R#” in Figure 4.

The above data in Figure 4 help to motivate the drug kinetics of the system (presented in Section 3.4) by showing the ability of the polymers to maintain their mass while they are immersed in an aqueous environment for an extended time period. 

### 3.4. Drug Release Kinetics

By varying the concentrations of Timolol in spectrophotometry, it was plausible to model a calibration curve. Concentrations of 0.005, 0.01, 0.025, and 0.05 mM were tested at the lowest spectrophotometer setting, 325 nm. This revealed a linear behavior, following Equation (2). From here, the Beer–Lambert law was utilized to determine the concentration, in mM, of Timolol in the system. 

**Figure 5:**
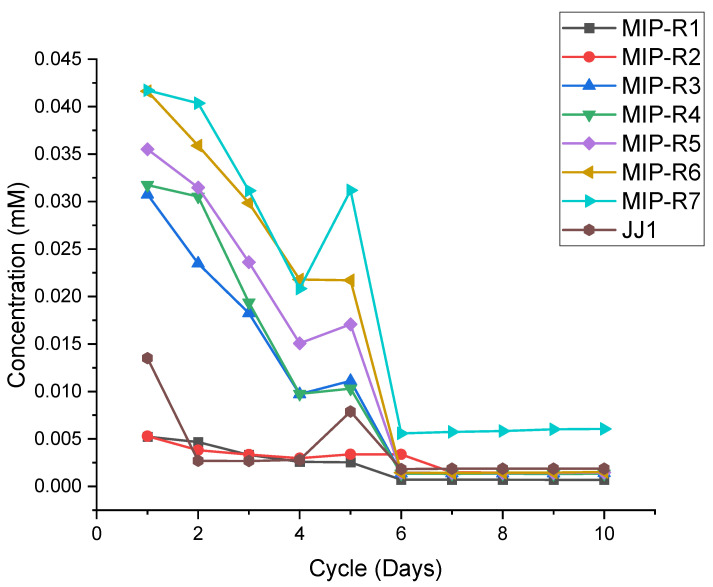
Demonstrating the MIP release and uptake kinetics, compared to JJ1. The concentration of Timolol, in mM, is shown on the *y*-axis. The *x*-axis demonstrates the cycle (in day) of measurement.

**Figure 6:**
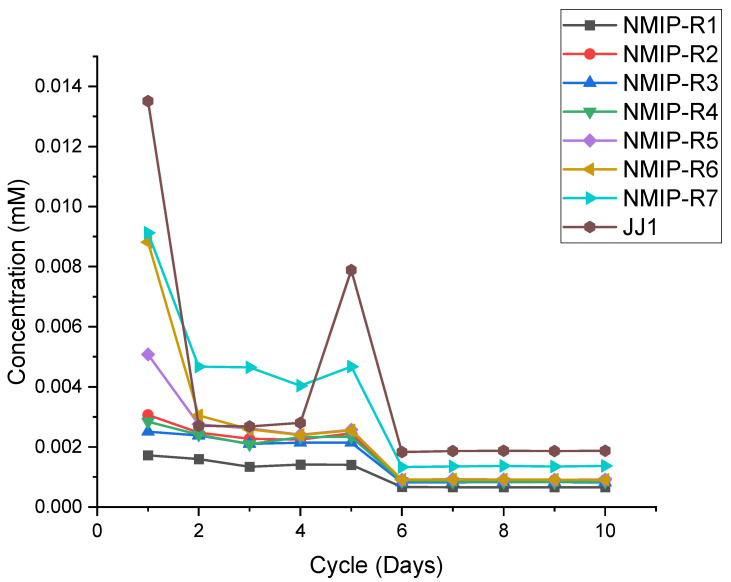
Demonstrating the NMIP release and uptake kinetics, compared to JJ1. The concentration of Timolol, in mM, is shown on the *y*-axis. The *x*-axis demonstrates the cycle (in day) of measurement.

Both MIP and NMIP samples were measured by using the same process, cycling between DI water and Timolol/DI solution every 24 h for a period of 10 days. This can be seen in Figure 5 (MIP) and Figure 6 (NMIP), below. MIP samples seem to show a higher concentration of Timolol throughout the experiment. Exhibited by Figure 5 are the initial high concentrations of Timolol in more densely crosslinked samples, MIP-R3 through MIP-R7, and the low concentrations from MIP-R1 and MIP-R2. 

All samples demonstrate a steady decline from this initial concentration until four days of being cycled. From here, a small increase is prevalent, followed by near-similar retention in the Timolol from day six to ten. Concentrations at days six through ten seem to propagate near 0.0025–0.010 mM of Timolol.

Figure 6 (below) demonstrates a similar idea, however, for NMIP samples. These were prepared and tested in the same manner, yet show varied results from the MIP samples. 

As demonstrated above by Figure 6, a similar phenomenon occurs with the decrease until day five, with steadiness in the data from day six to ten. The concentrations here are much lower than that seen by MIP samples in Figure 4, nearing 0.02 mM difference in some cases. JJ1, here, initially starts at 0.014 mM and decreases to 0.002 mM, which is far better retention than the NMIP samples. 

## 4. Discussion

The FTIR measurements conducted, as shown in Figure 2, demonstrate the key functional groups in the synthesized polymers. This characterization corresponds with the feed composition of the hydrogel being alkene-containing, due to the presence of acrylates added into the system through the backbone, crosslinker, and pH-sensitive monomer. The NMIP samples appear flat compared with the MIP samples, due to their inherent lack of hydrogen bonding between the Timolol and the polymer matrix. This corroborates the expected chemical composition of the HEMA hydrogel system. Henceforth, it is determinable that there will exist some sites for non-covalent bonding needed for drug-delivery MIP to take place. The optical properties are further demonstrated in Figure 3, where it is apparent that samples maintain high transparency of around 90–95% for the entire duration of the visible spectrum, which is 380–750 nm. Beyond this, they also exhibit decent performance beyond the visible spectrum to 300 nm, where it can be noticed that JJ1 samples exhibit a sharp decrease in their transmittance around 400 nm. 

The swelling ratios demonstrated in Figure 4 show a disparity between the more highly crosslinked samples and those with lower crosslinking density. Those samples with lower crosslinking are more prone to swelling, averaging a swelling ratio of 0.13, and overall network instability over the three days tested. This is in opposition to the higher crosslinked samples, which demonstrate a swelling ratio of only 0.07. Notably, this is combined with relative stability in the hydrogel, which only appears to have a maximum swelling ratio at three days, i.e., 0.04. Comparatively, the maximum swelling ratio at the same timestamp is 0.2. From this information, higher crosslinking furthers the overall stability of the hydrogel. This property is desirable in a hydrogel contact lens such as the idea proposed herein. Differences in the swelling ratio between that of JJ1 and samples R1–R4 are attributed to the chemical differences between these polymer constructions. While the precise composition of JJ1 is undeterminable, it appears to have a crosslinking density comparable to samples exhibiting higher swelling ratios, namely R5–R7. 

The drug-release kinetics demonstrated in Figure 5 and Figure 6 show a distinct disparity between the MIP and NMIP samples. MIP samples demonstrate a higher uptake volume than their NMIP counterparts, even for the same crosslinking density. This is expected due to the preformation of hydrogen-bonding sites within the polymer matrix. As these bond sites become preformed, the abilities for drug uptake and, thus, release are increased correspondingly. Higher densities of crosslinking, such as those seen in R4–R7 iterations, depict higher capabilities of drug uptake. This increased drug uptake volume corresponds with a higher release volume. Specifically, MIP-R7 contains near 0.05 mM of Timolol at 0.045 mM, meaning that it is more capable of delivering the required prescription amount. This is while the NMIP counterpart, NMIP-R7, contains only a fourth of this amount, nearing a peak of only 0.010 mM.

This demonstrated that the key to successful and prescription-comparable drug delivery is the use of MIP in the hydrogel system. Seen across all compositions, the NMIP samples have a noticeable maximum absorbance deficiency of 0.026 mM in some cases for the R6 and R7 compositions to a minimum of 0.002 mM in the R1 and R2 compositions. This is due to the lack of initial hydrogen-bonding sites within the material, meaning that the introduced Timolol must first bind itself in the polymerized environment. Specifically, the presence that NMIP-R2 exhibits higher control of its Timolol content for the duration of the test suggests that crosslinking in NMIP hydrogels deters these non-covalent bonding sites to some extent. A future direction here would be to identify the ability of JJ1 to perform in a MIP platform and compare it to the presented hydrogels. This is due to the lack of MIP-ready JJ1 samples, as they came polymerized.

The NMIP and MIP samples compared here exhibit a dependence on the crosslinking density, with the MIP platform being the most reliable platform for drug delivery. When the crosslinking density increases, as seen throughout the R1–R7 progression, the surface area increases within the pre-imprinted polymer. This alone allows for more bonding sites for the Timolol to bind itself to the hydrogel. However, in NMIP samples, some of the Timolol may be lost during the bonding process. By imprinting the Timolol, the individual monomers are forced to arrange themselves in a pattern conducive to the performance of the Timolol. Most importantly, it appears that the increase in the crosslinking density does not deter the hydrogel from optical performance, whereas, in Figure 3, it is determinable that there is low variance. The future of drug delivery will need to rely not only on the ability of high crosslinking to allow for drug uptake, but also on the controlled release of drugs out of the system. For this, it is hypothesized to study the effect of specific monomer components, outside of the crosslinker, on the ability of the hydrogel system to perform the controlled release. Combined with this study, one could develop a high-volume controllable system. This would allow for an efficient and consistent release of medication, as is more desirable for a commercial product.

## 5. Conclusions

In this paper, it was demonstrated how glaucoma could be treated by using a Timolol-containing drug-delivery vehicle. A HEMA-backbone hydrogel was synthesized due to its compatibility with Timolol. By varying the crosslinking density, we showed the dependency of Timolol release on the concentration of the crosslinker. Furthermore, it was shown that MIP hydrogels are more effective than their NMIP counterparts, especially in the highly crosslinked samples. MIP hydrogels promote the uptake and release of medication, while NMIP samples hamper the ability of the hydrogel to perform these tasks.

## Figures and Tables

**Table 1:** Composition changes in TEGDMA are expressed as the molar-weight fraction and molar-weight percentage of the entire volume. TEGDMA changes here from 1.11% of the system to 2.68% of the system throughout these compositions. The only change between NMIP and MIP samples is the addition of Timolol (x_i_ = 0.0005).

Composition	TEGDMA x_i_	TEGDMA x_i_%
R1	0.011	1.11
R2	0.014	1.43
R3	0.016	1.64
R4	0.019	1.91
R5	0.022	2.17
R6	0.024	2.44
R7	0.027	2.68

## Data Availability

Data can be made available from the authors upon reasonable request, utilizing the emails mentioned previously.
